# The Clinical Impact of Flow Titration on Epoprostenol Delivery via High Flow Nasal Cannula for ICU Patients with Pulmonary Hypertension or Right Ventricular Dysfunction: A Retrospective Cohort Comparison Study

**DOI:** 10.3390/jcm9020464

**Published:** 2020-02-07

**Authors:** Jie Li, Payal K. Gurnani, Keith M. Roberts, James B. Fink, David Vines

**Affiliations:** 1 Department of Cardiopulmonary Sciences, Division of Respiratory Care, Rush University Medical Center, Chicago, IL 60612, USA; kmr8106@sbcglobal.net (K.M.R.); fink.jim@gmail.com (J.B.F.); David_Vines@rush.edu (D.V.); 2 Department of Pharmacy, Rush University Medical Center, Chicago, IL 60612, USA; Payal_K_Gurnani@rush.edu; 3 Aerogen Pharma Corp, San Mateo, CA 94402, USA

**Keywords:** high-flow nasal cannula, epoprostenol, inhalation, flow titration, pulmonary hypertension

## Abstract

(1) Background: inhaled epoprostenol (iEPO) delivered via high-flow nasal cannula (HFNC) has been reported to be effective for pulmonary hypertension and right ventricular dysfunction. In vitro studies have identified HFNC gas flow as a key factor in trans-nasal aerosol delivery efficiency; however, little evidence is available on the clinical impact of flow titration on trans-nasal aerosol delivery. At our institution, iEPO via HFNC was initiated in 2015 and the concept of flow titration during iEPO via HFNC has been gradually accepted and carried out by clinicians in the recent years. (2) Methods: a retrospective review of the electronic medical records for all adult patients who received iEPO via HFNC in a tertiary teaching hospital. Pre- and post- iEPO responses were reported for patients whose HFNC flow was titrated or maintained constant during iEPO delivery. Positive response to iEPO was defined as the reduction of mean pulmonary arterial pressure (mPAP) > 10% for pulmonary hypertension patients or the improvement of oxygenation [pulse oximetry (SpO_2_)/fraction of inhaled oxygen (F_I_O_2_)] > 20%. The number of responders to iEPO was compared between groups with titrated vs constant flow. (3) Results: 51 patients who used iEPO to treat pulmonary hypertension and/or right ventricular dysfunction were reviewed. Following iEPO administration via HFNC, mPAP decreased (43.6 ± 11.7 vs. 36.3 ± 9.7 mmHg, *p* < 0.001). Among the 51 patients, 24 had concomitant refractory hypoxemia, their oxygenation (SpO_2_/F_I_O_2_) improved after iEPO delivery (127.8 ± 45.7 vs. 157.6 ± 62.2, *p* < 0.001). During iEPO initiation, gas flow was titrated in 25 patients and the remaining 26 patients used constant flow. The percentage of patients in the flow titration group who met the criteria for a positive response was higher compared to the group with constant flow (85.7% vs. 50%, *p* = 0.035). Pre- vs post-iEPO responses were significant in the flow titration group included improvement in cardiac output (*p* = 0.050), cardiac index (*p* = 0.021) and F_I_O_2_ reduction (*p* = 0.016). These improvements in hemodynamics and F_I_O_2_ were not observed in the constant flow group. (4) Conclusion: in patients with pulmonary hypertension and/or right ventricular dysfunction, trans-nasal iEPO decreased pulmonary arterial pressure. It also improved oxygenation in patients with combined refractory hypoxemia. These improvements were more evident in patients whose gas flow was titrated during iEPO initiation than those patients using constant flow.

## 1. Introduction

Inhaled epoprostenol (iEPO) is a pulmonary vasodilator which has been used off-label to treat pulmonary hypertension in the United States for over 15 years [1]. Inhaled epoprostenol has shown similar effectiveness in reducing pulmonary arterial pressure and improving oxygenation as inhaled nitric oxide (iNO) among mechanically ventilated patients [2,3,4,5,6,7]. Compared to iNO, iEPO is cost-saving and has become a common treatment in clinical practice. Due to its short half-life, iEPO is continuously delivered for extended periods of time (usually > 24 h), which becomes a challenge for spontaneous breathing patients, as conventional aerosol therapy via mouthpiece or mask for a long duration is uncomfortable and impossible to maintain [4]. Transnasal pulmonary delivery of iEPO via high-flow nasal cannula (HFNC) provides a feasible route, which has been shown to deliver sufficient dose to elicit a clinical response [8,9].

Multiple in vitro and in vivo scintigraphy studies have investigated influential factors of trans-nasal aerosol delivery and the ratio of gas flow to patient inspiratory flow was found to play a key role [10,11,12,13,14,15]. However, little evidence is available on its clinical impact. Additionally, no commercial device is currently available to monitor patient’s inspiratory flow on a breath by breath basis while receiving oxygen or ventilatory support. Therefore, the concept of flow titration during trans-nasal aerosol delivery based on clinical response may provide a surrogate to measuring a patient’s inspiratory flow [8]. As iEPO has a short onset time, titrating flow while monitoring a patient’s response is a pragmatic solution to identify the optimal flow. Since we started using iEPO via HFNC in our institution several years ago, this concept of titrating flow has been gradually accepted and carried out by clinicians. When iEPO was delivered via HFNC, gas flow was titrated based on patient’s response when clinicians were available. However, due to staff’s workload or availability, there were some patients still receiving constant flow during iEPO delivery via HFNC. This study was aimed at evaluating the clinical effects of flow titration during iEPO delivery via HFNC. Our hypothesis was that flow titration would deliver a higher inhaled dose of iEPO, which would increase the number of patients with a positive response to iEPO compared to those receiving constant flow.

## 2. Material and Methods

A retrospective review of all adult patients receiving iEPO via HFNC was conducted from July 2015 to October 2019, after approval by the institutional review board (approval No. 17062302-IRB01-CR03) in an academic institution in the United States (Rush University Medical Center). Adult patients ≥ 18 years and ordered to receive iEPO via HFNC to treat pulmonary hypertension/right ventricular dysfunction were enrolled. Pulmonary hypertension and right ventricular dysfunction were diagnosed by physicians and documented in medical record. Exclusion criteria included: (1) iEPO used for less than 30 min; (2) iEPO was used simultaneously with iNO; (3) concurrent systemic use of epoprostenol; (4) iEPO was initiated for palliative care; (5) iEPO was delivered via face mask; and (6) iEPO via HFNC was used immediately after extubation for patients who used iEPO during invasive ventilation.

### 2.1. The Process of iEPO Delivery via HFNC

Epoprostenol (Veletri, Actelion Pharmaceuticals US Inc, San Francisco, USA) (1.5 mg) was reconstituted with sterile water and prepared in a 50 mL syringe by pharmacy department. Once the order was prescribed, iEPO was initiated by a respiratory therapist at bedside and patient responses were recorded in the electronic medical record, following step-by-step instructions. The iEPO syringe was attached via tubing, to a vibrating mesh nebulizer (Aeroneb ^®^ Solo, Aerogen Ltd., Galway, Ireland) placed at the inlet of an active heated humidifier (MR 850, Fisher Paykel, New Zealand). A syringe pump was programmed to administer iEPO solution to the nebulizer at the rate prescribed. The initial dose of 50 ng/kg/min of iEPO was based upon ideal body weight, and maintained at this dose until weaned by 10 ng/kg/min as clinically appropriate per physician order [8].

Gas flow was initiated at the patient’s comfort level and maintained or titrated depending on the clinician’s availability. When gas flow was titrated, each flow was sustained for 10-15 min and patient’s clinical responses at each flow were recorded, with the final flow determined by the optimal physiologic response.

### 2.2. Data Collection

Patients’ demographic information including age, gender, race, documented systolic pulmonary arterial pressure (sPAP) by echocardiographic and right heart catheterization results [mean pulmonary arterial pressure (mPAP), cardiac output (CO), cardiac index (CI)] within 30 days prior to iEPO, diagnosis, and iEPO indication were collected. Patients’ responses pre and post iEPO were collected, including hemodynamics [heart rate (HR), mean blood pressure (mBP) or mean arterial pressure (MAP) if arterial line was placed, mPAP, CO and CI (if invasive hemodynamic monitoring was available)], and oxygenation [pulse oximetry (SpO_2_)/fraction of inhaled oxygen (F_I_O_2_)]. The use of vasopressors (drug and dose) and extracorporeal membrane oxygenation (ECMO) (if applicable) pre and post iEPO were also reviewed. Lastly, patient outcomes including iEPO duration, intubation, iEPO complications, survival and length of stay in ICU and hospital were collected.

The primary outcome was an observed improvement of hemodynamics, specifically mPAP, CO, and CI and oxygenation between two groups (flow titration vs constant flow). An mPAP reduction of > 10% for ≥ 5 min was defined as a positive response. For patients with concomitant refractory hypoxemia, defined as F_I_O_2_ ≥ 0.5 required to maintain SpO_2_ at 88‒93%. SpO_2_/F_I_O_2_ was substituted for PaO_2_/F_I_O_2_ to evaluate oxygenation [8,16] in cases where arterial blood gases before and after iEPO initiation were not available. A patient was also considered a responder to iEPO if SpO_2_/F_I_O_2_ increased by > 20% for ≥ 5 min or PaO_2_/F_I_O_2_ increased by > 20% within 60 min of iEPO initiation [8,9].

Secondary outcomes were intubation rate, iEPO duration and complications, ICU and hospital survival, ICU and hospital length of stay. Hemodynamic instability, defined as mBP reduction ≥ 20% or below 65 mmHg 30‒60 min after iEPO initiation, was counted as iEPO complication, as well as significant bleeding that required termination of iEPO. 

Due to the female predominance in many forms of pulmonary arterial hypertension [17], additional comparisons were implemented between female and male patients, as well as pre- vs. post- iEPO effects for female patients. 

### 2.3. Statistical Analysis

Kolmogorov–Smirnov was used to test normality of distribution for considered variables. Continuous variables were expressed as mean (standard deviation [SD]) or median (inter-quartile range [IQR]), depending on the normality of distribution. Pre and post iEPO variables were compared by paired t test or Wilcoxon sign rank in all patients, as well as in two groups (flow titration vs constant flow) separately, whereas the comparisons between two groups (flow titration vs constant flow) were analyzed by independent t test or Mann Whitney test. Differences in categorical variables were assessed using the chi-square test or fisher exact test. A *p*-value of < 0.05 was considered to be statistically significant for all tests. Data analysis was conducted with SPSS statistical software (SPSS 23.0 for windows; SPSS; Chicago, IL, USA).

## 3. Results

### 3.1. Demographic Information

**Figure 1:**
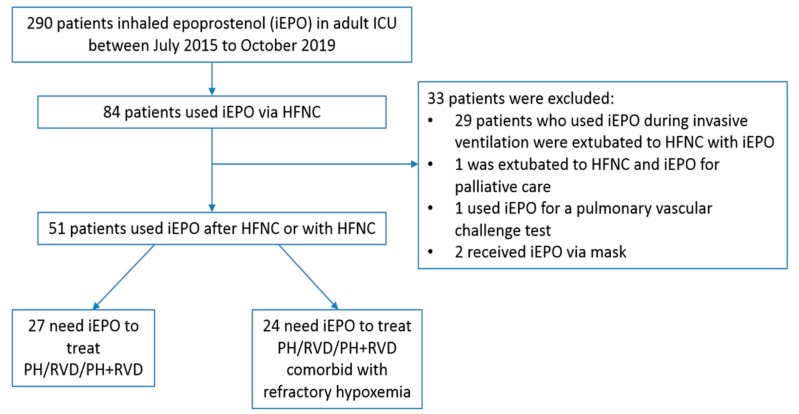
Study flowchart. iEPO, inhaled epoprostenol; HFNC, high-flow nasal cannula; PH, pulmonary hypertension; RVD, right ventricular dysfunction

Between July 2015 and October 2019, 290 adult ICU patients received iEPO, 84 of them receiving iEPO via HFNC. Thirty three patients were excluded due to the following reasons: 29 patients who used iEPO during invasive ventilation were extubated to HFNC with iEPO; 1 patient used iEPO less than 10 min; 1 patient used HFNC and iEPO for palliative care and 2 patients received iEPO via face mask (Figure 1). 

Among the remaining 51 patients receiving iEPO to treat pulmonary hypertension and/or right ventricular dysfunction, 22 (43.1%) were male and 26 (51%) were African Americans. Twenty-four (47.1%) patients had concomitant refractory hypoxemia. In the remaining 27 patients, 16 had pulmonary hypertension and 4 had right ventricular dysfunction, while 7 patients had both. Twenty-two patients used oxygen at home. Four patients were placed on ECMO during the time iEPO was initiated, with 2 of these patients cannulated on VA-ECMO and 2 patients cannulated on VV-ECMO (Table 1). Thirty-four patients had iEPO initiated after HFNC was used for 9.6 (0.96, 44.3) hours, while 17 patients started iEPO with HFNC simultaneously and only 1 of the 17 patients had concomitant refractory hypoxemia but didn’t respond. 

During iEPO initiation, iEPO dosage was maintained at 50 ng/kg/min for all patients. HFNC gas flow was titrated for 25 patients while the other 26 patients used constant flow. No significant differences were found in demographic information between the two groups, except for a larger number of patients in constant flow group with right ventricular dysfunction (57.7% vs. 24%, *p* = 0.023) (Table 1).

### 3.2. Pre- vs. Post- iEPO Hemodynamic Responses 

For all 51 patients, HR decreased from 97.2 ± 25.1 to 93.4 ± 19.7 bpm (*p* = 0.002), and mBP remained stable (*p* = 0.612). Invasive hemodynamic information which was available in 21 patients showed improvement after iEPO was delivered: mPAP decreased from 43.6 ± 11.7 to 36.3 ± 9.7 mmHg (*p* < 0.001), both CO (5.12 ± 1.81 vs. 6.11 ± 2.21 L/min, *p* = 0.024) and CI (2.65 ± 1.81 vs. 3.13 ± 0.98 L/min/m^2^, *p* = 0.011) were increased.

Gas flow was titrated based on mPAP in 13 patients, of whom 3 patients had concomitant refractory hypoxemia with iEPO initiated after HFNC, while 1 patient was placed on VA ECMO when iEPO was initiated. Gas flow with the lowest mPAP (optimal flow) was different for each individual (Table 2). In the flow titration group (*n* = 13), pre vs. post iEPO effects on mPAP, CO, CI and HR were significantly different. In contrast, in the constant flow group (*n* = 8), only pre vs post iEPO mPAP was significantly different (*p* = 0.030) while CO, CI and HR were unchanged (Table 3). Mean PAP reduction was higher in flow titration group than constant flow group (9.3 ± 6.3 vs. 4.2 ± 3.9 mmHg, *p* = 0.045, power = 0.517).

### 3.3. Pre- vs. Post- iEPO Oxygenation Responses for Patients Comorbid with Refractory Hypoxemia

For the 24 patients with concomitant refractory hypoxemia, iEPO was initiated in 23 of them after HFNC was used for 15.46 (2.46, 46.92) hours and SpO_2_/F_I_O_2_ significantly improved after iEPO was initiated (157.6 ± 62.2 vs. 127.8 ± 45.7, *p* < 0.001), with increased SpO_2_ (*p* = 0.008) and decreased F_I_O_2_ (*p* = 0.006). In the flow titration group (*n* = 12), gas flow was titrated from 50 (40, 50) L/min pre- iEPO to 30 (20, 40) L/min (*p* = 0.007) after iEPO was initiated. Pre- and post- iEPO SpO_2_/F_I_O_2_, SpO_2_ and F_I_O_2_ were significantly different. In contrast, in the group with constant gas flow at 40 (30, 45) L/min, only pre- and post- iEPO SpO_2_/F_I_O_2_ was significantly different (*p* = 0.034) while SpO_2_ and F_I_O_2_ were unchanged (Table 4).

### 3.4. Patient Outcomes

Using the specific criteria for positive response of mPAP reduction > 10% or SpO_2_/F_I_O_2_ improvement > 20% (for patients concomitant with refractory hypoxemia only), only 39 (76.5%) patients had a definite evaluation of their responses to iEPO. Among these 39 patients, 27 (69.2%) met the positive response criteria, while the percentage of responders was higher in the flow titration group than the constant flow group (85.7% vs. 50%, *p* = 0.035). Particularly, 6 of the 13 (46.2%) patients in the flow titration group had mPAP reduction > 20%, while no patients in the constant flow group had mPAP reduction > 20% (*p* = 0.046). Among the 27 responders, 15 had concomitant refractory hypoxemia, final gas flow was higher than in non-hypoxemic patients (32.0 ± 11.9 vs. 21.7 ± 11.5 L/min, *p* = 0.032). Moreover, 18 out of the 27 responders received flow titration and this percentage was higher than in non-responders (66.7% vs. 25%, *p* = 0.035).

No significant differences in iEPO duration, rate of intubation, ICU and hospital survival rates, and ICU and hospital length of stay were found between the flow titration and constant flow groups in all the 51 patients. Three patients had significant bleeding in the constant flow group, while no patients in the flow titration group had significant bleeding (Table 5). When the 4 ECMO patients were excluded from the analysis, the outcome of iEPO duration, intubation, ICU and hospital survival as well as length of stay between groups was similar (Supplemental Table S1).

For the 39 patients who had definite evaluation on iEPO responses, comparisons of demographic information and outcome between groups agreed with the findings aforementioned from the total 51 patients, except for the shorter ICU length of stay in flow titration group [10 (5.5, 14) vs. 13.5 (9.5, 21.8) days, *p* = 0.047], but the difference became insignificant after excluding the 3 ECMO patients (*p* = 0.089) (Supplemental Table S2).

For the 29 female patients, 16 used oxygen at home, this percentage was higher than male patients (55.2% vs. 27.3%, *p* = 0.046) (Supplemental Table S3.5). No difference of iEPO responders, intubation, ICU mortality was found between genders. Nine patients had invasive hemodynamic information that mPAP was significantly reduced after iEPO (*p* = 0.038) (Supplemental Table S3.1). Fifteen patients had concomitant refractory hypoxemia, pre- and post- iEPO oxygenation (SpO_2_/F_I_O_2_) was significantly improved in patients receiving flow titration (Supplemental Table S3.2).

## 4. Discussion

To our knowledge, this is the first study to report the clinical impact of flow titration on trans-nasal pulmonary aerosol delivery and the largest study to investigate the clinical effectiveness of iEPO delivery via HFNC. In this study, we found that iEPO delivery via HFNC reduced mPAP and improved CO and CI in patients with pulmonary hypertension or right ventricular dysfunction. Inhaled EPO also improved oxygenation in patients with concomitant refractory hypoxemia. More importantly, these benefits were found to be more evident among patients whose gas flow administered by HFNC was titrated during iEPO initiation than those receiving constant flow.

### 4.1. Clinical Impact of Flow Titration for iEPO Delivery via HFNC

In our previous study using iEPO delivery via HFNC at constant flow to treat severe hypoxemia in patients with pulmonary hypertension or right ventricular dysfunction, 5 out of 11 patients had a SpO_2_/F_I_O_2_ improvement of more than 20% [8], which is consistent with our response rate in the hypoxemic patients using constant flow. In this study, however, we found a trend of higher response rate in the group with flow titration (63.6% vs. 38.5%) (Table 5). Similarly, Ammar et al. reported a mPAP reduction of 4.9 ± 6.2 mmHg after iEPO via HFNC in 9 patients with pulmonary hypertension using constant flow [9], this finding is consistent with the constant flow group (4.2 ± 3.9 mmHg) but lower than the mPAP reduction in the flow titration group (9.3 ± 6.3 mmHg) in our study. Additionally, the percentage of responders who had mPAP reduction of greater than 20% was higher in the flow titration group (46.2% vs. 0, *p* = 0.046). These findings demonstrate that flow titration during iEPO initiation via HFNC improves the clinical responses to iEPO. This might be explained by the increased inhaled dose of iEPO delivered by titrated flow rather than constant flow [12,13], as iEPO has linear dose-response relationship [5].

Our in vitro studies found the inhaled dose of iEPO was higher when gas flow was set below the patient’s inspiratory flow [12,13]. The optimal delivery of iEPO occurred at a gas flow around 50% of patient’s inspiratory flow [12]. Since patients’ inspiratory flow varies by gender, height and disease state [18], this may explain why optimal flow for each patient was different. Since technology to measure a patient’s inspiratory flow during HFNC treatment is lacking, the optimal flow was based on the patient’s clinical response. Some of the patients in our flow titration group were evaluated at only a few specific gas flow rates, their response at lower or higher flows is unknown. A more complete assessment of all flows, especially low flows for non-hypoxemic patients, may achieve better hemodynamic and oxygenation responses and hopefully better outcomes.

### 4.2. Clinical Effects of iEPO Delivery via HFNC for Patients with Pulmonary Hypertension and/or Right Ventricular Dysfunction

Previous studies using iEPO via HFNC enrolled patients with severe hypoxemia and pulmonary hypertension and/or right ventricular dysfunction [8,9]. The primary outcome in these studies was improvement in oxygenation. In one of these studies, only a small percentage (25%) of the patients had invasive hemodynamic monitoring during iEPO initiation [9], which limited the assessment of iEPO’s impact on hemodynamics. In our study, 27 patients received HFNC treatment specifically for iEPO administration, not for oxygenation purpose. While this use of HFNC for delivering iEPO only has not been reported before, it has become a popular delivery route at our institution due to its comfort and feasibility of administration for extended periods of drug inhalation. HFNC functioned as the “vehicle” to carry continuous aerosolized medication for these patients [19]. As such, gas flow settings served primarily for aerosol delivery without affecting oxygenation or work of breathing [20]. Moreover, for these non-hypoxemic patients, breathing patterns are usually stable and inspiratory flow demand is low, so the HFNC gas flow setting should be low to achieve the optimal ratio of trans-nasal pulmonary aerosol delivery.

In contrast, for patients with concomitant refractory hypoxemia, inspiratory flow demands are high; thus, optimal gas flow for trans-nasal aerosol delivery should be higher than non-hypoxemic patients. More importantly, flow settings play an important role in oxygenation and work of breathing in those patients [19]. When iEPO is delivered via HFNC, a decreased gas flow may increase the inhaled dose of iEPO which improves oxygenation through improving ventilation perfusion match. When gas flow is set below patient inspiratory flow, the actual F_I_O_2_ inhaled will be lower due to the air entrainment and vary with changes in patient inspiratory flows [18,21]. These two effects seem contradictory on the patient’s oxygenation status; however, there is an optimal point at which both effects achieve the best oxygenation for the patient. This point may not be the same optimal point that benefits the trans-nasal pulmonary aerosol delivery for non-hypoxemic patients, and it needs to be determined through individual response at different flows. In general, we found that the final gas flow settings were higher in those patients with concomitant refractory hypoxemia than non-hypoxemic patients (32.0 ± 11.9 vs. 21.7 ± 11.5 L/min, *p* = 0.032).

In the 24 patients with concomitant refractory hypoxemia, final gas flow settings in the flow titration group were lower than the constant flow group (30 L/min (20, 40) vs. 40 L/min (30, 45)). Thus, the calculated/reported SpO_2_/F_I_O_2_ was lower than true value in the flow titration group, due to air entrainment; while the calculated SpO_2_/F_I_O_2_ was closer to the true value in the constant flow group. Despite this flaw, the reported SpO_2_/F_I_O_2_ was still higher in the flow titration group than the constant flow group (173.6 ± 73.1 vs. 141.6 ± 46.7). This finding emphasizes the advantages of flow titration in improving iEPO delivery.

### 4.3. Safety

The incidence of hemodynamic instability 30‒60 min after iEPO initiation in our study was lower than in the Ammar et al. study (15.7% vs. 39%) [9], which may be due to the definition of hemodynamic instability used. The incidence of significant bleeding was similar between the two studies (5.9% vs. 6%) [9]. All three patients that had significant bleeding in our study were in the constant flow group. Future studies are warranted to determine the exact rationale. More importantly, identifying responders and early termination of iEPO use in those who did not respond may be a better solution to avoid the potential risks or complications, as well as reduce associated expenses.

### 4.4. Limitations

There were several limitations in this study. It was not a prospective randomized controlled trial designed for comparing the clinical impacts of flow titration vs constant flow on iEPO delivery via HFNC, and the power for the analysis on the two groups was not sufficient, due to the small sample size. As such, clinical randomized controlled trials with larger sample size are warranted to confirm our findings.

Not all of the patients in the flow titration group had the same range of data to evaluate clinical effects, due to a lack of invasive monitoring techniques, such as a pulmonary artery catheter. Particularly those patients who required iEPO to treat pulmonary hypertension and/or right ventricular dysfunction, but were not hypoxemic, a lack of data on mPAP made titration difficult to perform. Future prospective studies with better evaluation tools are warranted to guide flow titration in these patients.

Similarly, not all patients received the full range of flow settings during titration. For example, several patients were initiated at 30 L/min then titrated down, while others started at 50 L/min and ended at 30 L/min, which may be due to reasons such as patient tolerance, preference, or clinician availability. Even though the number of patients with missing data points was not large, it was still possible that the “optimal flow” determined may have differed. Future studies should attempt to evaluate a full range of flow rates.

As more clinicians gained experience on titrating gas flow for patients during iEPO initiation, some of the flow settings in the constant flow group tended to be lower than in the past [8]. For example, several cases started flow directly at 20 L/min, which was unusual in conventional use of HFNC. Although the flow was unchanged during iEPO delivery, the lower flow settings increased the possibility delivering more iEPO. Thus, we assume our comparisons between the flow titration group and constant flow group may be more significant if the flow in the constant flow group was set at the usually higher setting.

In our clinical practice, we often use iEPO for severe hypoxemic patients without pulmonary hypertension or right ventricular dysfunction during mechanical ventilation [5,6,7]. We do not have experience with iEPO delivery via HFNC in this population before mechanical ventilation. Future studies are needed to investigate the effects of iEPO via HFNC for this specific population as well as the clinical impact of flow titration.

## 5. Conclusions

For patients with pulmonary hypertension and/or right ventricular dysfunction, trans-nasal inhalation of epoprostenol decreased pulmonary arterial pressure. If these patients also had concomitant refractory hypoxemia, oxygenation also improved. These improvements were more evident in patients whose gas flow rates were carefully titrated based on clinical response during iEPO initiation.

## Figures and Tables

**Table 1:** Comparisons of demographic information for patients with flow titration and constant flow.

	Overall	Flow Titration Group	Constant Flow Group	*p*
No. of patients	51	25	26	
Age, years	61.9 ± 16.7	63.4 ± 15.3	60.4 ± 18.0	0.529
Gender (Female) (%)	29 (56.9%)	13 (52 %)	16 (61.5%)	0.577
Race (%)				
African American	26 (51.0%)	12 (48.0%)	14 (53.8%)	0.716
Caucasian	18 (35.3%)	10 (40.0%)	8 (30.8%)	
Hispanic or Latino	6 (11.8%)	3 (12.0%)	3 (11.5%)	
Asian	1 (2.0%)	0	1 (3.8%)	
SICU (%)	23 (45.1%)	13 (52%)	10 (38.5%)	**0.592**
MICU (%)	15 (29.4%)	6 (24%)	9 (34.6%)	
Diagnosis (%)				0.112
PH	16 (31.4%)	10 (40%)	6 (23.1%)	
PH + Hypoxemia	14 (27.5%)	9 (36%)	5 (19.2%)	
PH + RVD	7 (13.7%)	3 (12%)	4 (15.4%)	
RVD	4 (7.8%)	0	4 (15.4%)	
RVD + Hypoxemia	4 (7.8%)	2 (8.0%)	2 (7.7%)	
PH + RVD + Hypoxemia	6 (11.8%)	1 (4%)	5 (19.2%)	
PH WHO classification				
Class I	21 (41.2%)	8 (32.0%)	13 (50.0%)	0.258
Class II	22 (43.1%)	14 (56.0%)	8 (30.8%)	0.093
Class III	16 (31.4%)	7 (28.0%)	9 (34.6%)	0.764
Class IV	3 (5.9%)	2 (8.0%)	1 (3.8%)	0.610
Class V	2 (3.9%)	2 (8.0%)	0	0.235
iEPO indication (%)				
PH	43 (84.3%)	23 (92%)	20 (76.9%)	0.248
RVD	21 (41.2%)	6 (24%)	15 (57.7%)	0.023
Hypoxemia	24 (47.1%)	12 (48%)	12 (46.2%)	1.0
iEPO initiated after HFNC (%)	34 (66.7%)	20 (80%)	14 (53.8%)	0.075
ECMO while iEPO was initiated (%)	4 (7.8%)	1 (4%)	3 (11.5%)	0.061
Chronic pulmonary disease (%)	22 (43.1%)	12 (48%)	10 (38.5%)	0.577
Home oxygen use (%)	22 (43.1%)	8 (32%)	14 (53.8%)	0.072
Baseline sPAP by Echo, mmHg ^a^	64 (50, 82.5)	61.8 ± 20.8	77.7 ± 28.7	0.058
Baseline mPAP, mmHg ^b^	45.7 ± 12.4	42.4 ± 6.8	51.7 ± 18.2	0.404
Baseline CO, L/min ^c^	4.93 ± 2.02	4.53 ± 1.25	5.53 ± 2.87	0.607
Baseline CI, L/min/m^2 c^	2.48 ± 1.04	2.20 ± 60	2.91 ± 1.43	0.328
Code status of do-not-intubate (%)	7 (13.7%)	4 (16%)	3 (11.5%)	0.703
Invasive hemodynamic monitoring available (%)	21 (41.2%)	13 (52.0%)	8 (30.8%)	0.124

iEPO, inhaled epoprostenol; HFNC, high-flow nasal cannula; MICU, medical intensive care unit; SICU, surgical intensive care unit; PH, pulmonary hypertension; RVD, right ventricular dysfunction; WHO, world health organization; ECMO, extracorporeal membrane oxygenation; mPAP, mean pulmonary arterial pressure; sPAP, systolic pulmonary arterial pressure; CO, cardiac output; CI, cardiac index. ^a^ Data available in 20 and 17 patients, respectively; ^b^ data available in 11 and 6 patients, respectively; ^c^ data available in 9 and 6 patients.

**Table 2:** Individual patient’s mPAP changes to different HFNC gas flows in the flow titration group of patients who had invasive hemodynamic monitoring.

Patient No.	iEPO Indication	F_I_O_2_ Prior to iEPO	Flow Prior to iEPO	mPAP (mmHg) at Different Gas Flow								Final Flow	Final F_I_O_2_	HFNC hours Prior to iEPO
				Prior to iEPO	50 L/min	40 L/min	30 L/min	20 L/min	10 L/min	5 L/min	At final Flow			
1	PH + hypoxemia	1.0	40	64.3	53	53	54.7	56.3			53	40	1.0	90
2	PH	0.3	30	56.7			50.7	47.7	42.7		42.7	10	0.3	0.33
3	PH	0.4	50	54.3	49.3	50.3	50.0	44.7	46.7		44.7	20	0.4	0.42
4	PH + hypoxemia	0.95	40	53.3	41.7	40.7	37	38.3			37	30	0.47	18.42
5 ^a^	PH	0.21	60	45.7	44.3	43.7	44.3	43	39.3	38.7	38.7	5	0.21	0.17
6	PH	0.4	50	44	39	36	35	40			34	30	0.4	1.85
7	PH + hypoxemia	0.5	50	41.3	41.3	37	33.3	30.3			32	20	0.5	6.33
8	RVD	0.4	30	22.3	21	20.3	20	19.3	17.7		17.7	10	0.4	1.08
9	PH	0.21	NA	65.3			56	55.3	55.3		55.3	20	0.3	0
10	PH	0.29	NA	42.7			21.7	20.0	20.3		20.0	10	0.3	0
11	PH	0.37	NA	43.0		38.7	40.7				38.7	40	0.4	0
12	PH	0.25	NA	44.7			40.7	43.7			40.7	30	0.25	0
13	RVD	0.29	NA	NA	22.3	21.3	21	20.7	19.7		19.7	10	0.35	0

PH, pulmonary hypertension; RVD, right ventricular dysfunction; mPAP, mean pulmonary arterial pressure; iEPO, inhaled epoprostenol; HFNC, high-flow nasal cannula. ^a^ patient was on VA ECMO when iEPO was initiated.

**Table 3:** Comparison of hemodynamic responses pre and post iEPO for all patients.

	No. of Patients	Prior to iEPO	Post iEPO	*p*	Power
mPAP, mmHg	21	43.6 ± 11.7	36.3 ± 9.7	<0.001	0.823
Flow titration group	13	46.9 ± 12.0	37.7 ± 10.8	0.002	0.741
Constant flow group	8	38.2 ± 9.4	34.0 ± 7.6	0.030	0.215
CO, L/min	14	5.12 ± 1.81	6.11 ± 2.21	0.024	0.380
Flow titration group	8	5.06 ± 1.79	6.55 ± 1.68	0.050	0.526
Constant flow group	6	5.20 ± 1.99	5.52 ± 2.83	0.462	0.057
CI, L/min/m^2^	15	2.65 ± 1.81	3.13 ± 0.98	0.011	0.213
Flow titration group	9	2.61 ± 0.78	3.29 ± 0.75	0.021	0.623
Constant flow group	6	2.71 ± 0.85	2.88 ± 1.30	0.416	0.061
HR, beats/min	51	97.2 ± 25.1	93.4 ± 19.7	0.002	0.210
Flow titration group	25	97.8 ± 22.2	93.2 ± 17.4	0.045	0.191
Constant flow group	26	96.7 ± 28.0	93.6 ± 22.0	0.198	0.091
mBP, mmHg	51	82.5 ± 15.4	81.7 ± 14.4	0.612	
RR, breaths/min	51	20.9 ± 5.8	20.4 ± 5.6	0.305	
F_I_O_2_	51	0.5 (0.33, 0.8)	0.45 (0.35, 0.6)	0.065	
SpO_2_, %	51	95 (91, 98)	97 (94, 99)	0.030	
SpO_2_/F_I_O_2_	51	212.8 ± 99.9	218.2 ± 85.4	0.345	

iEPO, inhaled epoprostenol; HFNC, high-flow nasal cannula; mPAP, mean pulmonary arterial pressure; CO, cardiac output; CI, cardiac index; HR, heart rate; RR, respiratory rate; mBP, mean blood pressure; F_I_O_2_, fraction of inhaled oxygen; SpO_2_, pulse saturation of oxygenation.

**Table 4:** Comparison of parameters and oxygenation responses pre and post iEPO for 24 patients with concomitant refractory hypoxemia.

	No. of Patients	Prior to iEPO	Post iEPO	*p*	Power
SpO_2_/F_I_O_2_	24	127.8 ± 45.7	157.6 ± 62.2	<0.001	0.705
Flow titration group	12	133.4 ± 52.5	173.6 ± 73.1	0.003	0.493
Constant flow group	12	122.2 ± 39.1	141.6 ± 46.7	0.034	0.283
F_I_O_2_	24	0.8 (0.53, 1.0)	0.65 (0.5, 0.89)	0.006	
Flow titration group	12	0.79 ± 0.23	0.64 ± 0.25	0.016	0.483
Constant flow group	12	0.79 ± 0.20	0.72 ± 0.20	0.140	0.190
SpO_2_, %	24	92.5 (89, 96)	95.5 (93, 97.8)	0.008	
Flow titration group	12	92.5 ± 4.3	95.9 ± 2.5	0.037	0.844
Constant flow group	12	89.9 ± 8.8	94.0 ± 3.9	0.098	0.454
HFNC flow, L/min	23	40 (40, 50)	40 (30, 40)	0.007	
Flow titration group	11	50 (40, 50)	30 (20, 40)	0.007	
Constant flow group	12	40 (30, 45)	40 (30, 45)	NA	
HFNC duration, hours	24	15.5 (2.5, 46.9)	NA		
HR, beats/min	24	102.0 ± 28.3	94.2 ± 19.8	0.018	
mBP, mmHg	24	85.3 ± 13.9	83.8 ± 13.9	0.549	
mPAP, mmHg	6	47.5 ± 11.3	40.4 ± 7.5	0.116	
RR, breaths/min	24	23.0 ± 6.2	22.6 ± 6.2	0.542	

iEPO, inhaled epoprostenol; HFNC, high-flow nasal cannula; HR, heart rate; RR, respiratory rate; mBP, mean blood pressure; F_I_O_2_, fraction of inhaled oxygen; SpO_2_, pulse saturation of oxygenation.

**Table 5:** Comparisons outcome for patients receiving titrated flow and constant flow.

	Overall	Flow Titration	Constant Flow	*p*
No. of patients	51	25	26	
Responders in total ^a^	69.2% (27/39)	85.7% (18/21)	50% (9/18)	0.035
Responders of hypoxemic patients using criteria of SpO_2_/F_I_O_2_ improvement > 20%	50% (12/24)	63.6% (7/11)	38.5% (5/13)	0.414
Responders using criteria of mPAP reduction > 10%	76.2% (16/21)	84.6% (11/13)	62.5% (5/8)	0.325
Responders with mPAP reduction > 20%	28.6% (6/21)	46.2% (6/13)	0	0.046
iEPO duration, hours	64.5 (34.8,107.3)	55 (37, 101.7)	69.7 (22.9, 165.5)	0.843
Intubation (*n*,%)	8 (15.7%)	2 (8%)	6 (23.1%)	0.248
iEPO complications (*n*,%)	11 (21.6%)	4 (16%)	7 (26.9%)	0.214
Bleeding	3 (5.9%)	0	3 (11.5%)	
Hemodynamic instability	8 (15.7%)	4 (16%)	4 (15.4%)	
ICU alive (*n*,%)	40 (78.4%)	21 (84%)	19 (73.1%)	0.499
ICU stay, days	11 (6, 18)	10 (6, 14)	11.5 (6.8, 19.5)	0.257
Hospital alive (*n*,%)	37 (72.5%)	19 (76%)	18 (69.2%)	0.755
Hospital stay, days	13 (8, 20)	12 (7, 18.5)	14 (9.3, 21.5)	0.509

iEPO, inhaled epoprostenol; SpO_2_, pulse saturation of oxygenation; F_I_O_2_, fraction of inhaled oxygen; mPAP, mean pulmonary arterial pressure; ICU, intensive care unit. ^a^ data available in 21 and 18 patients, respectively.

## Supplementary Materials

The following are available online at https://www.mdpi.com/2077-0383/9/2/464/s1, Table S1: Non-ECMO patients’ outcome, Table S2: Comparisons of demographic information and outcome with titrated flow vs constant flow for patients who had definite evaluation on responses, Table S3.1: Comparison of hemodynamic responses pre and post iEPO for female patients, Table S3.2: Comparison of parameters and oxygenation responses pre and post iEPO for female patients, Table S3.3: Comparison of hemodynamic responses pre and post iEPO for male patients, Table S3.4: Comparison of parameters and oxygenation responses pre and post iEPO for male patients, Table S3.5: Comparison of demographic information and outcome between male and female patients, Table S4.1: Response to iEPO via HFNC between patients with and without type I pulmonary hypertension, Table S4.2: Comparison of oxygenation response to iEPO via HFNC using flow titration versus constant flow in patients with type I pulmonary hypertension.
