# Supplementary material for: The Clinical Impact of Flow Titration on Epoprostenol Delivery via High Flow Nasal Cannula for ICU Patients with Pulmonary Hypertension or Right Ventricular Dysfunction: A Retrospective Cohort Comparison Study
Source: J Clin Med. 2020 Feb 7;9(2):464. doi: 10.3390/jcm9020464 (PMC7074129; doi:10.3390/jcm9020464)
Supplement: Supplementary file 1 [file jcm-09-00464-s001.pdf]

Supplemental Table S1. Non-ECMO patients' outcome.

	Overall	Flow titration	Constant flow	*p*
No. of patients	47	24	23	
iEPO duration, hours	64.5 (34.8,104.6)	59.8 (36, 104.5)	68.4 (21.6, 104.6)	0.873
Intubation (%)	6 (12.8%)	2 (8.3%)	4 (17.4%)	0.416
ICU alive (%)	37 (78.7%)	20 (83.3%)	17 (73.9%)	0.494
ICU length of stay, days	10 (6, 15)	9.5 (6, 14)	11 (6, 17)	0.257
Hospital alive (%)	34 (72.3%)	18 (75%)	16 (69.6%)	0.752
Hospital length of stay (days)	12 (7, 17)	12 (6.5, 16.8)	12 (7,17)	0.915

iEPO, inhaled epoprostenol; ICU, intensive care unit; ECMO, extracorporeal membrane oxygenation.

Supplemental Table S2. Comparisons of demographic information and outcome with titrated flow vs constant flow for patients who had definite evaluation on responses.

	Overall	Flow titration	Constant flow	*p*
No. of patients	39	21	18	
Age, years	62.7 ± 17.5	63.4 ± 16.4	59.9 ± 20.1	0.555
Gender (Male) (%)	19 (48.7%)	12 (57.1%)	7 (38.9%)	0.341
Race: African American (%)	18 (46.2%)	9 (42.9%)	9 (50%)	0.664
SICU (%)	21 (53.8%)	12 (57.1%)	9 (50%)	0.905
MICU (%)	8 (20.5%)	4 (19%)	4 (22.2%)	
Diagnosis (%)				0.033
PH	11 (28.2%)	9 (42.9%)	2 (11.1%)	
PH + Hypoxemia	14 (35.9%)	9 (42.9%)	5 (27.8%)	
PH + RVD	1 (2.6%)	0	1 (5.6%)	
RVD	3 (7.7%)	0	3 (16.7%)	
RVD + Hypoxemia	4 (10.3%)	2 (9.5%)	2 (11.1%)	
PH + RVD+ Hypoxemia	6 (15.4%)	1 (4.8%)	5 (27.8%)	
iEPO indication (%)				
PH	32 (82.1%)	19 (90.5%)	13 (72.7%)	0.215
RVD	14 (35.9%)	3 (14.3%)	11 (61.1%)	0.003
Hypoxemia	24 (61.5%)	12 (57.1%)	12 (66.7%)	0.742
ECMO while iEPO was initiated (%)	3 (7.1%)	1 (4.8%)	2 (11.1%)	0.586
Chronic pulmonary disease (%)	17 (43.6%)	10 (47.6%)	7 (38.9%)	0.748
Home oxygen use (%)	15 (38.5%)	6 (28.6%)	9 (50%)	0.134
sPAP by Echo, mmHg [b]	63 (49, 82.5)	59.8 ± 22.3	80.8 ± 28.7	0.141
mPAP, mmHg [c]	45.6 ± 6.6	44.6 ± 5.4	50.5 ± 12.2	NA
CO, L/min [d]	4.40 ± 2.14	4.42 ± .73	6.55 ± 3.04	NA
CI, L/min/m^2 [d]	2.47 ± 1.01	2.14 ± .26	3.65 ± 2.06	NA
Responders (%)	27 (69.2%)	18 (85.7%)	9 (50%)	0.035
iEPO duration (hours)	55 (30.7, 107.3)	53.1 (37, 101.7)	70.7 (20.2, 165.5)	0.967
Intubation (%)	6 (15.4%)	2 (9.5%)	4 (22.2%)	0.387
iEPO Complications (%)	10 (25.6%)	4 (19%)	6 (33.3%)	0.150
Bleeding	3 (7.7%)	0	3 (16.7%)	
Hemodynamic instability	7 (17.9%)	4 (19%)	3 (16.7%)	
ICU alive (%)	31 (79.5%)	18 (85.7%)	13 (72.2%)	0.432
ICU stay, days	12 (8, 16)	10 (5.5, 14)	13.5 (9.5, 21.8)	0.047
Non-ECMO patients [e]		9 (5.3, 13.8)	12 (8.5, 18.5)	0.089
Hospital alive (%)	29 (74.4%)	17 (81%)	12 (66.7%)	0.465
Hospital stay (days)	13 (8, 20)	12 (7, 16.5)	14 (11, 23.3)	0.269

iEPO, inhaled epoprostenol; HFNC, high-flow nasal cannula; PH, pulmonary hypertension; RVD, right ventricular dysfunction; ECMO, extracorporeal membrane oxygenation; EF, ejection fraction; mPAP, mean pulmonary arterial pressure; sPAP, systolic pulmonary arterial pressure; CO, cardiac output; CI, cardiac index; ICU, intensive care unit. [a] data available in 17 and 12 patients, respectively; [b] data available in 16 and 9 patients, respectively; [c] data available in 9 and 2 patients, respectively; [d] data available in 7 and 2 patients, respectively; [e] data available in 20 and 16 patients, respectively.

Supplemental Table 3.1 Comparison of hemodynamic responses pre and post iEPO for female patients.

	No. of patients	Prior to iEPO	Post iEPO	*p*
mPAP, mmHg	9	44.7 ± 8.5	38.8 ± 4.6	0.038
Flow titration	4	46.5 ± 10.0	38.9 ± 4.3	0.144
Constant flow	5	43.3 ± 8.0	38.7 ± 5.3	0.138
CO, L/min	7	4.93 ± 2.01	5.53 ± 2.06	0.351
Flow titration	3	5.53 ± 2.79	6.37 ± 1.76	0.593
Constant flow	4	4.48 ± 1.49	4.90 ± 2.28	0.461
CI, L/min/m^2	7	2.51 ± 0.91	2.89 ± 1.03	0.207
Flow titration	3	2.43 ± 1.07	2.97 ± 0.64	0.285
Constant flow	4	2.58 ± .93	2.83 ± 1.36	0.414

Supplemental Table 3.2 Comparison of parameters and oxygenation responses pre and post iEPO for female patients.

	No. of patients	Prior to iEPO	Post iEPO	*p*
SpO_2/F_IO_2	15	128.6 ± 50.0	163.5 ± 73.7	0.003
Flow titration	7	145.14 ± 57.2	196.9 ± 85.1	0.028
Constant flow	8	114.0 ± 41.0	134.2 ± 50.5	0.069
F_IO_2	15	0.79 ± 0.22	0.68 ± 0.25	0.042
Flow titration	7	0.74 ± 0.24	0.59 ± 0.29	0.066
Constant flow	8	0.84 ± 0.20	0.77 ± 0.20	0.279
SpO_2, %	15	90.5 ± 8.1	95.4 ± 3.2	0.028
Flow titration	7	92.4 ± 4.0	96.0 ± 2.8	0.150
Constant flow	8	88.8 ± 10.4	94.9 ± 3.6	0.107

Supplemental Table 3.3 Comparison of hemodynamic responses pre and post iEPO for male patients.

	No. of patients	Prior to iEPO	Post iEPO	*p*
mPAP, mmHg	12	42.7 ± 13.9	34.4 ± 12.1	0.002
Flow titration	9	47.1 ± 13.3	37.1 ± 12.9	0.008
Constant flow	3	29.5 ± 2.0	26.2 ± 1.0	0.102
CO, L/min	7	5.31 ± 1.72	6.69 ± 2.35	0.063
Flow titration	5	4.78 ± 1.20	6.66 ± 1.83	0.080
Constant flow	2	NA	NA	NA
CI, L/min/m^2	8	2.76 ± 0.70	3.34 ± 0.95	0.035
Flow titration	6	2.70 ±0 .70	3.45 ± 0.80	0.046
Constant flow	2	NA	NA	NA

Supplemental Table 3.4 Comparison of parameters and oxygenation responses pre and post iEPO for male patients.

	No. of patients	Prior to iEPO	Post iEPO	*p*
SpO_2/F_IO_2	9	126.5 ± 40.3	147.9 ± 38.1	0.028
Flow titration	5	117.0 ± 45.9	141.1 ± 39.7	0.043
Constant flow	4	138.5 ± 34.2	156.4 ± 39.9	0.465
F_IO_2	9	0.79 ± 0.21	.68 ± 0.19	0.063
Flow titration	5	0.86 ± 0.22	0.72 ± 0.19	0.102
Constant flow	4	0.70 ± 0.18	0.63 ± 0.19	0.317
SpO_2, %	9	92.4 ± 4.4	94.2 ± 3.6	0.168
Flow titration	5	92.6 ± 5.2	95.8 ± 2.4	0.104
Constant flow	4	92.3 ± 4.0	92.3 ± 4.3	1.0

Supplemental Table 3.5 Comparison of demographic information and outcome between male and female patients.

	Male	Female	*p*
No. of patients	22	29	
Age, years	67.4 ± 14.3	57.7 ± 17.3	0.039
Race			0.182
African American (%)	8 (36.4%)	18 (62.1%)	
Caucasian	10 (45.5%)	8 (27.6%)	
SICU (%)	13 (59.1%)	10 (34.5%)	0.054
MICU (%)	7 (31.8%)	8 (27.6%)	
Diagnosis (%)			0.065
PH	10 (45.5%)	6 (20.7%)	
PH + Hypoxemia	5 (22.7%)	9 (31%)	
PH + RVD	0 (13.7%)	7 (24.1%)	
RVD	3 (13.6%)	1 (3.4%)	
RVD + Hypoxemia	1 (4.5%)	3 (10.3%)	
PH + RVD + Hypoxemia	3 (13.6%)	3 (10.3%)	
iEPO indication (%)			
PH	18 (81.8%)	25 (86.2%)	0.713
RVD	7 (31.8%)	14 (48.3%)	0.266
Hypoxemia	9 (40.9%)	15 (51.7%)	0.573
ECMO while iEPO was initiated (%)	2 (9.1%)	2 (6.9%)	1.0
Chronic pulmonary disease (%)	8 (36.4%)	14 (48.3%)	0.569
Home oxygen use (%)	6 (27.3%)	16 (55.2%)	0.046
Code status of do-not-intubate (%)	2 (9.1%)	5 (17.2%)	0.684
Intubation	4 (18.2%)	4 (13.8%)	0.713
ICU mortality	4 (18.2%)	7 (24.1%)	0.737
Hospital mortality	6 (27.3%)	8 (27.6%)	1.0
Responder (39)	14/19 (73.7%)	13/20 (65.0%)	0.731

iEPO, inhaled epoprostenol; HFNC, high-flow nasal cannula; MICU, medical intensive care unit; SICU, surgical intensive care unit; PH, pulmonary hypertension; RVD, right ventricular dysfunction; ECMO, extracorporeal membrane oxygenation.

Supplement table 4.1 Response to iEPO via HFNC between patients with and without type I pulmonary hypertension.

	Type I PH	Other types of PH	*p*
mPAP reduction (%)[a]	19.6 (6.4, 27.6)	16.2 (11.7, 22.1)	0.399
Responders with mPAP reduction > 10%	80% (4/5)	11/12 (91.7%)	0.515
Responders with mPAP reduction > 20%	40% (2/5)	33.3% (4/12)	1.0
SpO_2/F_IO_2 improvement for patients with concomitant refractory hypoxemia (%)[b]	17.8 (4.1, 51.2)	16.3 (0.5, 37.3)	0.564
Responders of hypoxemic patients using criteria of SpO_2/F_IO_2 improvement > 20%	50% (7/14)	50% (3/6)	1.0

iEPO, inhaled epoprostenol; SpO_2, pulse saturation of oxygenation; F_IO_2, fraction of inhaled oxygen; mPAP, mean pulmonary arterial pressure; PH, pulmonary hypertension. [a]Data was available in 5 and 12 patients with PH type I and other types, respectively. [b]Data was available in 14 and 6 patients with PH type I and other types, respectively.

Supplement table 4.2 Comparison of oxygenation response to iEPO via HFNC using flow titration versus constant flow in patients with type I pulmonary hypertension.

	Flow titration	Constant flow	*p*
SpO_2/F_IO_2 improvement for patients with concomitant refractory hypoxemia (%)[a]	35.6(9.9, 161.4)	13.1 (0, 43.0)	0.257
Responders of hypoxemic patients using criteria of SpO_2/F_IO_2 improvement > 20%	60% (3/5)	44.4% (4/9)	1.0

iEPO, inhaled epoprostenol; SpO_2, pulse saturation of oxygenation; F_IO_2, fraction of inhaled oxygen; mPAP, mean pulmonary arterial pressure; PH, pulmonary hypertension. [a]Data was available in 5 and 9 patients with flow titration and constant flow, respectively.
